# No change in prevalence of symptoms of COPD between 1996 and 2006 in Finnish adults – a report from the FinEsS Helsinki Study

**DOI:** 10.3402/ecrj.v3.31780

**Published:** 2016-08-16

**Authors:** Annette Kainu, Paula Pallasaho, Anne Pietinalho

**Affiliations:** 1 HUCH Heart and Lung Center, Peijas Hospital, Helsinki University Hospital and University of Helsinki, Helsinki, Finland; 2 Espoo City Health Services, Espoo, Finland; 3 Raasepori Health Center, Raasepori, Finland; 4 Finnish Lung Health Association, Helsinki, Finland

**Keywords:** smoking, obstructive airways disease, chronic bronchitis, epidemiology, dyspnoea

## Abstract

**Background:**

The age-dependent increase of chronic obstructive pulmonary disease (COPD) prevalence caused by smoking and other inhalational exposures in the general population is well-known worldwide. However, time trends are poorly known, due to lower number of high-quality studies especially following nationwide efforts on diminishing exposure levels. This study aimed to compare the prevalence of COPD symptoms and their major determinants in Finnish adults in 1996 and 2006.

**Methods:**

Two identical postal surveys were conducted among two random population samples from Helsinki using identical methodologies in 1996 and 2006, with 6,062 (76%) and 2,449 (62%) participants, respectively.

**Results:**

The physician-diagnoses of COPD remained at 3.7%, whereas physician-diagnoses of asthma and use of asthma medicines increased in both genders. Current smoking reduced from 33.4 to 27.3% (*p*<0.001), and the amount of cigarettes smoked also reduced significantly. The crude prevalence of chronic productive cough was 12.1 and 11.1%, wheezing with dyspnoea without a cold (wheezing triad) 7.3 and 7.7%, and dyspnoea grade II 13.8 and 13.6%, in 1996 and 2006, respectively. Among subjects with physician-diagnosed COPD, the prevalences of chronic productive cough and recurrent wheeze reduced significantly, from 60.6 to 40.7% and 53.5 to 38.5%, respectively.

**Conclusion:**

From 1996 to 2006, the prevalence of obstructive airway symptoms common in different phenotypes of COPD did not increase in Finnish adults. This suggests that the upward trend of COPD prevalence might have reached a plateau. Current smoking and the quantities smoked diminished suggesting a wider impact of stronger legislation and smoking-cessation efforts during the Finnish National Programme for COPD.

Chronic obstructive pulmonary disease (COPD) is globally a major public health problem and one of the leading causes of morbidity, disability, and death (1, 2). The most important cause of COPD is smoking, but other inhalational exposures, genetic and predisposition, and childhood infections have also been implicated with a significant role in the development of COPD (2–4). The disease is frequently unrecognised, underdiagnosed, and undertreated, and is associated with chronic symptoms that reduce the quality of life even before the development of airflow limitation that lays the foundation for current diagnostic criteria for COPD (3, 5). Epidemiological studies using different diagnostic criteria for chronic airflow limitation have reported increasing prevalence estimates and future projections from various countries (6–11), but interestingly some studies have also shown stable (Finland and England) or even reducing prevalences (Spain and the USA) (12–15). COPD is not an unidimensional disease, but instead encompasses different phenotypes, shares common features, and coexists with chronic asthma in adults (2, 16, 17). Diagnostic criteria from the international guidelines advocate the use of spirometry in confirming the diagnosis of not fully reversible airways obstruction (18). These spirometric criteria do not fully reflect the phenotypes of chronic bronchitis, emphysema, and frequent exacerbators, and the use of Global Initiative for Chronic Obstructive Lung Disease (GOLD) criteria in spirometry is debated (3, 18–25). Thus, use of repeated and validated symptom-based surveys is also valuable in the assessment of prevalence COPD in a general population (3, 20). Comparisons of the prevalence figures between cohorts are acceptable when published symptom patterns typical for COPD are defined (26).

In the prevalence of asthma, a plateau has been suggested (27), but despite growing evidence there is less recognition for similar levelling off for COPD possibly because varying diagnostic methods have made comparisons less conclusive (12–15). From earlier studies, we have shown that subjects with incident COPD were symptomatic even 10 years prior to diagnosis, thus typical respiratory symptoms could predict smokers potentially at risk of developing COPD (28).

COPD is widely underdiagnosed, and thus, it is assumed that estimates of its prevalence using physician-diagnoses are significantly affected by the functionality of the primary health care system (28–32). Changes in smoking habits and respiratory symptoms common in COPD could be less dependent on the changes in health care practices and diagnostic activity.

The aim of the present study was to compare the prevalence of physician-diagnosed COPD and related respiratory symptoms and their major determinants, smoking, age, and sex, in Finnish adults in 1996 and 2006 to assess time trends in prevalence.

## Materials and methods

The FinEsS study is a joint Nordic study on clinical respiratory epidemiology between Finland, Estonia, and Sweden that began in 1996. The study protocol has been reported earlier (33). In summary, both population samples have been randomly selected from the national population register stratified by sex and 10-year age cohorts between 20 and 69 years using identical sampling protocols selecting 8,000 subjects in Finland in 1996 and 4,000 subjects in 2006. Subjects that had died or moved outside Helsinki before the questionnaire mailing were excluded yielding a final sample of 7,990 and 3,968 subjects in 1996 and 2006, respectively. In total, 6,062 subjects (3,462 women) or 76% responded in 1996 and 2,449 subjects (1,365 women) or 62% responded in 2006. In the 2006 study, response rate fell especially in younger women as reported earlier (33).

### Ethics statement

The study protocol has been reviewed by the Ethics Board of the Helsinki University Hospital.

### Questionnaire and definitions

The FinEsS postal questionnaire was originally developed for the first Swedish Obstructive Lung Disease in Northern Sweden (OLIN) study in 1985 from a revised version of the British Medical Research Council questionnaire (34, 35). The study questionnaire has been published previously (36). The postal questionnaires used were identical for the first 15 questions. Only questions relevant to this paper are outlined.

*Chronic productive cough*: Relates to affirmative answers to three consecutive questions of ‘Do you usually have phlegm when coughing, or do you have phlegm which is difficult to bring up?’, ‘Do you bring up phlegm on most days during periods of at least three successive months?’, and ‘Have you had such periods during at least two successive years?’.

*Recurrent wheeze*: ‘Have you had wheezing, whistling, or a noisy sound in your chest when breathing?’

*Wheeze 12 months*: ‘Have you had wheezing or whistling in the chest at any time in the last 12 months?’

*Wheeze with dyspnoea*: ‘Have you been at all breathless when the wheezing sound was present?’

*Wheeze without cold*: ‘Did you have wheezing or whistling when you didn't have a cold?’

*Wheeze triad*: Positive answers all three questions of wheeze 12 months, wheeze with dyspnoea and wheeze without cold.

*Dyspnoea grade II*: ‘Do you get short of breath walking with other people of your own age on level ground?’

*Exercise provoking symptoms*: ‘Do you usually have breathlessness, wheeze, or severe cough on effort?’

*Physician-diagnosis of asthma*: ‘Have you been diagnosed with asthma by a doctor?’

*Physician-diagnosis of COPD*: ‘Have you been diagnosed with COPD, chronic bronchitis or emphysema by a doctor?’

*Use of asthma medicines*: ‘Do you currently use asthma medicines (permanently or as needed)?’

*Self-reported allergic rhinoconjunctivitis (arc)*: ‘Have you now or have you had any of the following diseases? Allergic eye-/nose catarrh (hay fever)’

*Symptoms suggestive of COPD* refer to chronic productive cough, recurrent wheeze, wheeze triad, exercise provoking symptoms, and dyspnoea grade II.

*Current smoker* refers to those currently or within 1 year having been smoking any quantity of cigarettes, pipe, or cigars. *Former smokers* are those who report previous smoking, but who have quit smoking at least 1 year previously. *Ever smokers* include all current and former smokers, and *non-smokers* include only subjects who have never smoked.

### Statistical analyses

For prevalence comparisons, the two-sided chi-squared test was used to analyse differences between groups, and Mantel–Haenzel test was used for trends in age and smoking categories. Prevalence analyses were stratified for age, gender, and smoking history. For risk analyses, multiple logistic regression was used to calculate odds ratios (OR) and 95% confidence intervals (CI). All statistical analyses were conducted using Statistical Package for Social Sciences (IBM Corp. Released 2013. IBM SPSS Statistics for Macintosh 22.0, Armonk, NY, USA). *P*-values less than 0.05 were considered significant in all analyses.

## Results

### Trends in prevalence 1996–2006

Physician-diagnosed COPD was reported in 3.7% of responders both in 1996 and 2006 with a non-significant gender difference (Table 1). The prevalence and frequency of smoking decreased in both genders with the prevalence of current smokers diminishing from 37.2 to 30.9% in men (*p*=0.001) and 29.7 to 22.7% in women (*p*<0.001). In addition to a reduced proportion of responders reporting current smoking, those smoking also reported smoking smaller quantities of cigarettes with the largest reduction in the group of >15 cigarettes/day in men from 18.7 to 12.7% in 1996 and 2006, respectively (*p*<0.001).

**Table 1:** Prevalence (%) of respiratory symptoms and of smoking in 1996 and 2006 by sex

	Male		Female		All	
			
	1996 (%)	2006 (%)	1996 (%)	2006 (%)	1996 (%)	2006 (%)
Age						
<40 years	**44.7**	**39.1**	**46.0**	**38.9**	**45.4**	**39.0**
40–59 years	**41.9**	**41.9**	**41.4**	**41.2**	**41.6**	**41.6**
≥60 years	**13.4**	**19.0**	**12.6**	**19.9**	**13.0**	**19.5**
Smoking						
Non-smoker	**39.9**	**42.9**	**54.9**	**55.2**	**48.4**	**49.7**
Former smoker	**22.8**	**26.3**	**15.5**	**22.1**	**18.6**	**24.0**
Current smoker	**37.2**	**30.9**	**29.7**	**22.7**	**32.9**	**26.3**
Current smoking intensity (current smokers)						
None	**0.8**	**3.2**	1.1	4.0	**0.9**	**3.6**
<5 cigarettes/day	**18.1**	**27.7**	24.0	25.1	**21.1**	**26.5**
5–14 cigarettes/day	**34.1**	**31.2**	43.9	42.2	**39.1**	**36.6**
≥15 cigarettes/day	**46.9**	**37.9**	31.1	28.5	**38.8**	**33.3**
Use of asthma medication	**4.6**	**7.7**	**7.0**	**10.7**	**6.0**	**9.4**
Previous asthma	**6.1**	**9.7**	**8.0**	**11.3**	**7.2**	**10.6**
Allergic rhinoconjunctivitis	**32.8**	**38.7**	**39.9**	**45.9**	**36.9**	**42.7**
Physician-diagnosis of asthma	**5.6**	**9.3**	**7.3**	**10.6**	**6.6**	**10.0**
Physician-diagnosis of COPD	4.1	3.5	3.5	3.9	3.7	3.7

Statistical significance *p*<0.05 is indicated by bold font.

The crude prevalences of respiratory symptoms suggestive of COPD remained unchanged, with only wheeze in the last 12 months in women increasing significantly. Chronic productive cough even decreased significantly in women from 11.7% in 1996 to 9.6% in 2006. Dyspnoea grade II and the wheeze triad remained unchanged during 10 years (Table 2). Symptoms suggestive of COPD, namely chronic productive cough, wheeze triad, recurrent wheeze, and dyspnoea grade II, show very little if any difference between 1996 and 2006, when stratified by gender, age, smoking history, and current smoking intensity (Table 2).

**Table 2:** Prevalence (%) of chronic productive cough, recurrent wheeze, wheeze triad, and dyspnoea grade II stratified by age, sex, and smoking history, in 1996 and 2006, respectively

	Chronic productive cough			Recurrent wheeze			Wheeze triad			Dyspnoea grade II		
				
	1996 (%)	2006 (%)	*P*	1996 (%)	2006 (%)	*P*	1996 (%)	2006 (%)	*P*	1996 (%)	2006 (%)	*P*
All	12.1	11.1	0.188	7.3	7.2	0.914	7.3	7.7	0.535	13.8	13.6	0.726
Sex												
Female	11.7	9.6	0.036	6.5	6.7	0.832	7.4	7.6	0.763	16.3	15.1	0.294
Male	12.7	13.0	0.794	8.3	7.9	0.672	7.3	7.8	0.551	10.5	11.6	0.339
Age												
<40 years	9.1	8.7	0.692	5.3	5.1	0.804	6.5	9.0	0.010	7.3	5.9	0.123
40–59.9 years	13.0	11.3	0.165	8.1	7.9	0.792	7.5	7.2	0.742	16.5	15.1	0.305
≥60 years	19.8	15.5	0.053	11.5	10.1	0.444	9.7	6.3	0.036	28.0	25.6	0.349
Smoking												
Non-smoker	8.0	6.9	0.223	3.9	3.4	0.504	4.9	5.6	0.340	10.4	8.6	0.083
Former smoker	11.9	11.1	0.625	7.1	7.3	0.855	8.0	8.0	0.980	17.4	18.6	0.534
Current smoker	18.3	19.1	0.677	12.4	14.3	0.225	10.6	11.5	0.521	16.9	18.3	0.427
Current smoking intensity												
None	9.2	7.9	0.119	4.6	4.1	0.337	5.7	6.0	0.668	12.1	11.6	0.599
<5 cigarettes/day	7.3	9.0	0.457	4.3	8.5	0.030	5.8	11.5	0.011	9.2	8.5	0.758
5–14 cigarettes/day	13.6	15.0	0.564	10.3	10.5	0.932	9.5	10.1	0.751	14.9	18.0	0.230
≥15 cigarettes/day	27.4	31.8	0.182	18.6	25.5	0.018	13.6	14.2	0.814	23.8	27.2	0.277

**Fig. 1:**
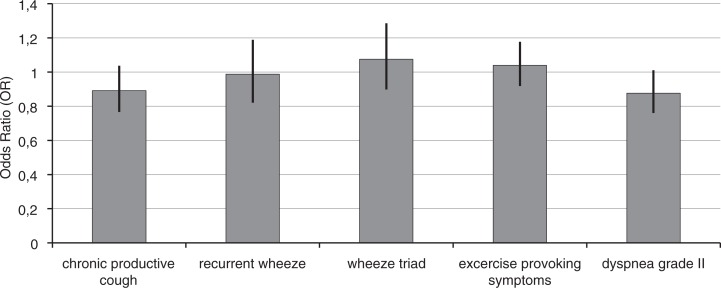
Adjusted odds ratios of study year 2006 versus 1996 for prevalence of symptoms suggestive of COPD. Multiple logistic regression analysis was performed with adjustment for age, sex, and smoking history (non, former, and current). Ninety-five percent confidence intervals are included.

Almost all of the COPD-related symptoms remained unchanged between 1996 and 2006 when age, gender, and smoking history were taken into consideration (Fig. 1). Chronic productive cough (OR 0.89; 95% CI 0.77–1.04), wheeze triad (1.07; 0.90–1.29), and dyspnoea grade II (OR 0.88; 0.76–1.01) did not show significant changes, whereas more asthma-related symptoms of shortness of breath in 12 months (OR 1.22; 1.07–1.40) and wheeze during 12 months (OR 1.21; 1.08–1.37) increased slightly during the 10-year interval.

### COPD and chronic productive cough with previous asthma

**Fig. 2:**
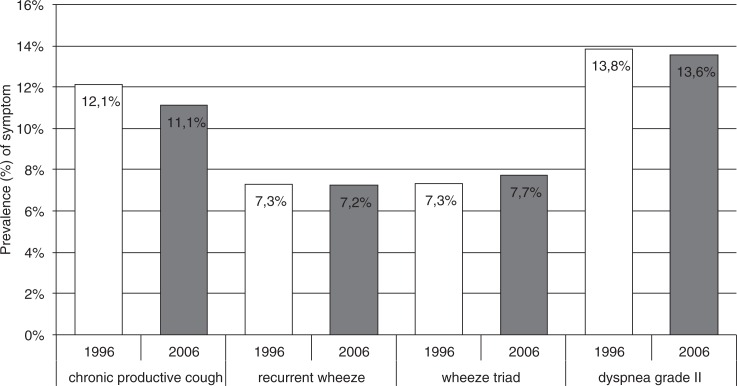
Prevalence (%) of respiratory symptoms suggestive of COPD in 1996 and 2006 in the Finnish general population.

**Fig. 3:**
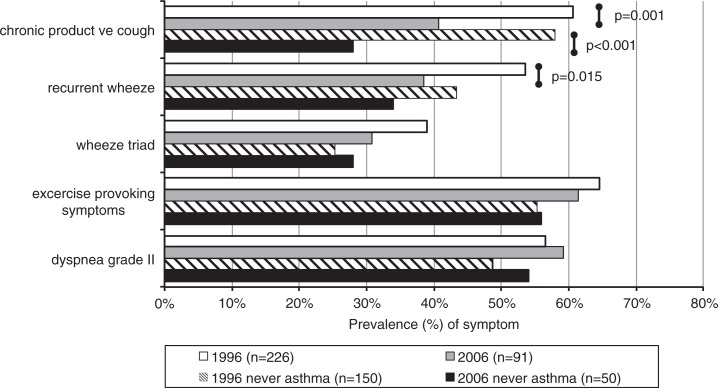
Prevalence (%) of respiratory symptoms suggestive of COPD in subjects with physician-diagnosed chronic obstructive pulmonary disease (COPD) in the general Finnish population in the 1996 and 2006 surveys stratified by previous history of asthma.

Of the subjects with physician-diagnosed COPD, 30.5 and 46.2% reported having a concurrent diagnosis of asthma (*p*=0.008) and 58.0 and 61.5% of allergic rhinoconjunctivitis (*p*=0.558), in 1996 and 2006, respectively. Use of asthma medication was reported by 33.2% of subjects with physician-diagnosed COPD in 1996, which had increased up to 49.5% in the 2006 study responders (*p*=0.007). During the same time interval, the subjects reporting physician-diagnosed asthma reported using asthma medication from 71.1 to 70.3%. The prevalence of COPD symptoms stratified by previous asthma shows a slight inverse trend in COPD-related symptoms in asthmatics. The overall prevalences of symptoms suggestive of COPD in the population remained unchanged as shown in Fig. 2. In 2006, symptoms were clearly more prevalent in subjects with concurrent diagnosis of asthma and slightly more prevalent in subjects with allergic rhinoconjunctivitis. Allergic rhinoconjunctivitis and previous asthma would seem to predispose towards more symptoms also in smokers. However, among subjects with physician-diagnosed COPD, the prevalences of chronic productive cough and wheeze with dyspnoea reduced significantly from 60.6 to 40.7% (*p*=0.001) and 50.0 to 37.4% (*p*=0.019) in 1996 and 2006, respectively (Fig. 3). The decrease in chronic productive cough was more pronounced among subjects without concurrent self-reported asthma.

### Risk factor analysis

In the multiple regression model using study year, sex, age, smoking, and self-reported allergic rhinoconjunctivitis as independent variables, chronic cough, and dyspnoea grade II were significantly less prevalent, and the use of asthma medication was significantly more prevalent in 2006 compared to 1996. All symptoms suggestive of COPD increased with age, reported current smoking, and the presence of allergic rhinoconjuncitivitis (Table 3).

**Table 3:** Risk factors, including study year, for chronic productive cough, recurrent wheeze, dyspnoea grade II, and use of asthma medication by multiple logistic regression analysis

	Chronic productive cough		Recurrent wheeze		Wheeze triad		Dyspnoea grade II		Use of asthma medicines	
					
	OR (95% CI)	*P*	OR (95% CI)	*P*	OR (95% CI)	*P*	OR (95% CI)	*P*	OR (95% CI)	*P*
Study year 2006 (vs. 1996)	**0.83 (0.71**–**0.97)**	**0.019**	0.91 (0.75–1.10)	0.307	0.97 (0.81–1.17)	0.751	**0.84 (0.72**–**0.97)**	**0.015**	**1.44 (1.21**–**1.72)**	**<0.001**
Male sex	1.13 (0.99–1.30)	0.073	**1.23 (1.04**–**1.46)**	**0.019**	1.02 (0.86–1.21)	0.808	**0.58 (0.51**–**0.67)**	**<0.001**	**0.72 (0.60**–**0.86)**	**<0.001**
Age (vs. <40 years)										
40–59.9 years	**1.60 (1.37**–**1.87)**	**<0.001**	**1.79 (1.47**–**2.17)**	**<0.001**	1.14 (0.95–1.37)	0.169	**2.74 (2.33**–**3.21)**	**<0.001**	1.15 (0.94–1.40)	0.169
≥60 years	**3.01 (2.48**–**3.66)**	**<0.001**	**3.12 (2.44**–**3.97)**	**<0.001**	**1.53 (1.20**–**1.96)**	**0.001**	**6.24 (5.17**–**7.53)**	**<0.001**	**2.06 (1.62**–**2.61)**	**<0.001**
Smoking (vs. never)										
Former	**1.41 (1.17**–**1.72)**	**<0.001**	**1.74 (1.35**–**2.23)**	**<0.001**	**1.63 (1.29**–**2.05)**	**<0.001**	**1.86 (1.57**–**2.20)**	**<0.001**	**1.42 (1.12**–**1.77)**	**0.002**
Current	**2.98 (2.55**–**3.48)**	**<0.001**	**4.15 (3.39**–**5.08)**	**<0.001**	**2.45 (2.02**–**2.96)**	**<0.001**	**2.41 (2.07**–**2.80)**	**<0.001**	1.15 (0.94–1.41)	0.179
Allergic rhinoconjunctivitis	**2.41 (2.10**–**2.77)**	**<0.001**	**3.10 (2.61**–**3.69)**	**<0.001**	**4.57 (3.81**–**5.47)**	**<0.001**	**2.17 (1.90**–**2.47)**	**<0.001**	**5.65 (4.65**–**6.87)**	**<0.001**

All variables in the table were included in the model. Associations are presented as odds ratios (OR) with 95% confidence intervals (95% CI). Statistically significant associations in bold text.

## Discussion

This large general population-based repeated postal survey study found no further increase after 1996 in symptoms suggestive of COPD among Finnish adult residents in the Helsinki capital area. From 1996 to 2006, the study responders reported no increase in physician-diagnoses of COPD. However, it is known that considerable underdiagnosis and underrecognition occurs with COPD; and thus, we analysed the trends in prevalences of symptoms suggestive of COPD and found no increase in COPD-related symptoms in the general population. Among subjects with physician-diagnosed COPD, the prevalence of symptoms even slightly decreased. This study reinforces the finding that COPD prevalence has indeed reached a plateau with symptoms suggestive of COPD showing no significant changes in either gender.

### Trends in symptoms of COPD

The prevalence of COPD symptoms on average did not change during the 10 years in the general population. Chronic productive cough diminished in women, but this was related to less prevalent current smoking and after taking current smoking into account on a multivariate model, there were no significant changes. Previously, it has been suggested that females would be more likely to report breathlessness and cough, whereas males would be more likely to report phlegm (37). Our findings are consistent with these results showing lower reported prevalences of chronic productive cough and higher prevalences of dyspnoea grade II in women both in 1996 and 2006 with the prevalence of chronic productive cough decreasing significantly from 11.7 to 9.6% (*p*=0.036). Symptoms related to wheeze were on comparable levels in both genders. In the Mini-Finland Study from 1978 to 1980, chronic bronchitis was reported in 23.8 and 13.8% and dyspnoea grade II in 10.1 and 12.8% of men and women, respectively (38). In that study, half of the women reporting chronic bronchitis were lifelong never smokers. Also Lundbäck et al. have previously reported higher prevalence of chronic productive cough in never smoking women; and it has been hypothetised that women are more susceptible to smoking-related adverse effects on lung function and chronic bronchitis (39–41). In our study 38.2% of women and 24.1% of men reporting chronic productive cough in 2006 were non-smokers, the proportion of non-smokers did not change from 1996. The impacts of environmental tobacco exposure (ETS) and unrecognised asthma on the higher prevalences of chronic bronchitis in non-smoking women have not been fully evaluated. ETS has been shown to increase bronchial hyperreactivity (42). In both our surveys, chronic productive cough was assessed with exactly same format of questions in 1996 and 2006 as in the Mini Finland Study and showing a consistently reducing trend. Concurrently, implemented tobacco-control measures have reduced ETS exposure, and besides a true increase in asthma prevalence, the improved recognition of asthma in primary care has increased the proportion of diagnosed asthmatics from 1.4% in men and 2.2% in women during the Mini Finland Study to 9.3 and 10.6% in men and women, respectively, in our 2006 survey (39).

In Northern Finland, a similar survey was conducted in 1996, where chronic productive cough was found in 11.5% and dyspnoea grade II in 14.6% (43). The prevalence of smoking and ETS in Kemi was slightly higher. In 1996, chronic productive cough was reported in 5.6% in Stockholm, 11.7% in Helsinki, and 8.9% in Tallinn, whereas both long-standing cough and sputum production were more prevalent in Tallinn in line with more prevalent smoking in Estonia at the time (26). No prospective data are available from the other capitals to assess possible concurrent trends in prevalence at this stage.

Limited data exist of longitudinal evaluation of COPD symptoms in the general population, since most studies have followed cohorts of COPD patients over time (8, 44). In a longitudinal study on symptoms in a COPD cohort, the symptom reporting was similar by sex over 3 years (45). In severe COPD, symptoms may vary greatly over the day and the week, with breathlessness being the most common symptom (46). Dyspnoea has been found to be the most disabling symptom of COPD correlating more closely with health-related quality of life and decreased activity levels (47). In our study, the crude prevalence of dyspnoea grade II remained unchanged in both genders.

Undiagnosed COPD has been found to be associated with impaired health-related quality of life and activities of daily living. Symptoms are already more prevalent several years previously in subjects with incident COPD (5, 28, 44). The observed plateau in symptoms related to COPD would suggest that the true trend of prevalence of symptomatic COPD might remain at a steady state with no evidence to support the increases in COPD prevalence, morbidity, and mortality projected in other countries (1, 9, 11).

### Questionnaire data on screening and case finding of COPD

Several studies have evaluated the role of questionnaires in assessing the prevalence and detection of COPD. Barr et al. validated the self-reported COPD in a cohort of nurses and confirmed from medical records 78% of cases reporting a physician-diagnosis of COPD. It was suggested that questionnaire-based COPD research should focus on minimising false positives rather than false negatives (48). In general, true cases of COPD are often symptomatic if detailed questions of respiratory symptoms are evaluated, whereas subjects might not actively observe these symptoms or might relate them to continued smoking. From the Lung Function Questionnaire (LFQ), symptoms of wheeze, dyspnoea, and cough in addition to age and smoking, predicted best airflow obstruction consistent with COPD in the population (49). In another study, an overall LFQ score of 18 or less was found to have a relatively good sensitivity of 88%, but weaker specificity of 25%, thus showing potential for being used for screening subjects for further spirometry. Questionnaire data alone had a low positive predictive value but good negative predictive value (50).

### Trends in smoking

This repeated cross-sectional survey shows a declining trend both in current smoking and in the smoking intensity in both genders. The timing of our study relates to a nationwide change in anti-smoking legislation, which has further banned smoking in workplaces (from 1995) and restaurants (from 2003 to 2007) (51). The Finnish National COPD Programme from 1998 to 2007 also included efforts in provision of smoking cessation services and knowledge to the primary care (52). Concurrently new pharmaceutical products came into market providing new tools and also public interest in smoking cessation efforts. These might have contributed to the observed significant reduction in smoking prevalences. Despite the observed decrease in prevalence of smoking in women between 1996 and 2006, current smoking remained still higher than in the Mini Finland Study from 1978 to 1980. The prevalence of current smoking continued on a decreasing trend compared to both our 1996 survey and the results from the nationwide Health 2000 study (12, 38).

### Trends in physician-diagnoses of asthma and COPD

The physician-diagnoses of asthma increased during the observed time interval, which has been hypothetised to relate to better recognition of asthma in the primary care following the Finnish Asthma Programme 1994–2004 (33, 53). In the Mini Finland Health Survey, 1.4% men and 2.2% women reported previously diagnosed asthma, whereas 2.8% of men and 5.2% women had previous asthma in the Health 2000 survey (12, 38). In our study, the prevalence of self-reported physician-diagnosed asthma increased from 5.6 to 9.3% in men and 7.3 to 10.6% in women between the 1996 and 2006 surveys. It has also been reported that the diagnosed asthmatics were less symptomatic suggesting better asthma-control in the diagnosed subjects (33). However, physician-diagnoses of COPD remained unchanged during the same time period. It has been questioned whether or not this would be related to lack of recognition of COPD or possibly diagnostic mislabelling of COPD as asthma. The latter cannot be excluded by questionnaire data, but our results show that symptoms suggestive of symptomatic COPD in the population did not increase but instead key symptoms of chronic productive cough and dyspnoea grade II even decreased, when the confounding variables of gender, age, smoking history, and allergic rhinoconjunctivitis were taken into consideration (Table 3). The question of physician-diagnosed COPD includes chronic bronchitis, which is a symptom-based diagnosis, making our definition of physician-diagnosed COPD broader than strictly obstruction-based diagnosis of COPD. Current smoking continued to show a decreasing trend with both the number of subjects reporting to have smoked during the preceding 12 months and the amount currently smoked per day decreasing significantly both in men and women and in all age categories.

Globally rising trends of prevalences of obstruction and COPD have been reported and further increases projected (1–3, 6–9, 11, 32, 54). The diagnostic criteria used in lung function measurements result in caution comparing the results. Assessment of COPD prevalence using repeated cross-sectional studies with spirometry can also have biases with an unexpectedly large reduction in COPD prevalence observed in Spain, demonstrating also a wide difference in prevalence estimates depending on the criteria used (14). These difficulties of assessing COPD prevalence in cross-sectional studies raise the important issue of caution when making deductions from single studies evaluating the prevalence of complex disorders like COPD (14). Our findings are consistent with previously reported lung function data from the Health 2000 study in Finland and further reinforce the observation of a plateau in the prevalence of symptomatic COPD in Finland. These findings also provide further support to the continuing efforts of anti-tobacco measures and improved identification and active treatment of both asthma and COPD in the primary care.

### Strengths and limitations

The present study applied similar postal questionnaire surveys in 1996 and 2006 using identical sampling. The response rate in 2006 decreased especially in young female adults (33). COPD, however, is known to be a disease most prevalent in the older age cohorts and strongly associated with a prior history of smoking; thus, the decreasing response rate in young women could impact as a slight overestimation of COPD symptoms, thus strengthening our results of no increase in COPD-related symptoms. Another limitation is the format of the questionnaire survey, with no clinical data to verify the diagnoses of COPD or asthma. In the US, self-reported COPD could be confirmed by clinical data on medical records in 78% of the self-reported diagnoses in a study on nurses (48). From a nationally representative sample of the Health 2000 study, no change in the prevalence of airflow limitation consistent with COPD has been reported compared to the 1978–1980 Mini Finland study (12). Respiratory symptoms or concurrent diagnoses of asthma or allergic rhinoconjunctivitis were not assessed from that sample. The strengths of our study rise from using strictly controlled survey sampling and validated questionnaire methodology in two cohorts 10 years apart. The subjects were surveyed using their regular medication; thus, asthmatic subjects were evaluated during their regular treatment.

## Conclusion

In conclusion, the present study demonstrated that symptoms of COPD and chronic bronchitis have not increased in prevalence from 1996 to 2006 in Finnish adults. The observed decrease in current smoking and the amounts smoked may contribute to this finding in addition to improved recognition of asthma in primary care. This gives further support to the continuing efforts to target the recognised risk factors for COPD to eventually turn the tide in COPD prevalence and the rising health care and human costs related to this chronic condition.
